# Diacylglycerol kinase α promotes 3D cancer cell growth and limits drug sensitivity through functional interaction with Src

**DOI:** 10.18632/oncotarget.2344

**Published:** 2014-08-12

**Authors:** Pedro Torres-Ayuso, Manuel Daza-Martín, Jorge Martín-Pérez, Antonia Ávila-Flores, Isabel Mérida

**Affiliations:** ^1^ Department of Immunology and Oncology, Centro Nacional de Biotecnología/CSIC, Madrid, Spain; ^2^ Department of Cancer Biology, Instituto de Investigaciones Biomédicas Alberto Sols/CSIC, Universidad Autónoma de Madrid, Madrid, Spain

**Keywords:** Diacylglycerol kinase, Src, 3D tumor growth, chemotherapy resistance, PI3K/Akt

## Abstract

Diacylglycerol kinase (DGK)α converts diacylglycerol to phosphatidic acid. This lipid kinase sustains survival, migration and invasion of tumor cells, with no effect over untransformed cells, suggesting its potential as a cancer-specific target. Nonetheless the mechanisms that underlie DGKα specific contribution to cancer survival have not been elucidated. Using three-dimensional (3D) colon and breast cancer cell cultures, we demonstrate that DGKα upregulation is part of the transcriptional program that results in Src activation in these culture conditions. Pharmacological or genetic DGKα silencing impaired tumor growth *in vivo* confirming its function in malignant transformation. DGKα-mediated Src regulation contributed to limit the effect of Src inhibitors, and its transcriptional upregulation in response to PI3K/Akt inhibitors resulted in reduced toxicity. Src oncogenic properties and contribution to pharmacological resistance have been linked to its overactivation in cancer. DGKα participation in this central node helps to explain why its pharmacological inhibition or siRNA-mediated targeting specifically alters tumor viability with no effect on untransformed cells. Our results identify DGKα-mediated stabilization of Src activation as an important mechanism in tumor growth, and suggest that targeting this enzyme, alone or in combination with other inhibitors in wide clinical use, could constitute a treatment strategy for aggressive forms of cancer.

## INTRODUCTION

The diacylglycerol kinases (DGK) phosphorylate diacylglycerol (DAG) to produce phosphatidic acid (PA); DGK thus regulate the levels of two lipids with key roles in signaling and metabolic pathways [1]. Many reports support a positive role for the DGKα isoform in cancer progression. This lipid kinase assists tumor survival [2-4], migration and invasion [5-7]. DGKα expression is increased in tumors compared to adjacent non-cancerous tissue [3, 4], as well as in metastasis of the original tumor [8, 9]. When mutated, it is a causative gene in pancreatic cancer [10], and was recently described as a potential target for glioblastoma treatment [11]. Nonetheless, the mechanisms by which DGKα contributes to these processes, and the exact tumor context for which DGKα is an effective target have not been elucidated.

DGKα function is regulated by transcriptional and post-transcriptional mechanisms. Distinct routes converge and are integrated at the *DGKα* gene promoter region, including those of PI3K/Akt/FoxO, p53 and Ras [12-14]. DGKα is a cytosolic enzyme, and its phosphorylation by distinct members of the Src family kinases (SFK) lead to its recruitment to the plasma membrane and activation [15-18]. SFK are non-receptor tyrosine kinases that share a common modular structure including a SH3 and a SH2 domains involved in protein interactions, and a myristoylation site at the N-terminus for membrane targeting [19]. *In vitro* experiments with GST (glutathione S-transferase)-purified DGKα and recombinant Src mapped DGKα interactions with Src SH2 and SH3 regions [18].

Src is the most widely expressed member of the SFK family and is relevant in many cancer types, since it controls tumor cell proliferation, survival, migration and invasion [20, 21]. Src regulates mitogenic and survival signaling cascades downstream of receptors tyrosine kinase (RTK), which are frequently mutated and/or overexpressed in breast and colon cancer. Oncogenic Src functions are also related to its activation downstream of integrins to regulate survival and invasion [22]. Src activity is predictive of poor clinical prognosis in colon and pancreatic cancer [23, 24]. These findings have led to substantial efforts to test the therapeutic potential of Src inhibitors in advanced cancers such as breast and colon, which are very frequent tumor types and tend to present early relapse and metastasis. Although preclinical evidence supported the use of such inhibitors, its therapeutic effectiveness as single agents in clinical assays for solid tumors has been discouraging [25]. This is probably due to incomplete knowledge of the mechanisms that control Src transforming potential and of the cancer-related Src-regulated pathways. Src is involved in many fundamental cellular processes, but the Src deficient mice are viable [26]. In contrast to viral oncoproteins, Src alone is insufficient to transform cells *in vitro*, and activating mutations of SFK are rarely observed in cancer [27, 28], suggesting further control by specific Src regulators that promote or restrain its tumorigenic properties [29, 30].

Here we tested the hypothesis that DGKα expression in tumor cells contributes to oncogenic SFK functions. We analyzed the DGKα contribution to breast and colon cancer in 3D cell cultures. These culture conditions closely mimic the *in vivo* cell environment and have been used to demonstrate the activation of transcription programs that lead to tumor survival and drug resistance [31-33]. Tumor cell growth in 3D culture is particularly dependent on integrin and Src signaling cascades, a property that it is not recapitulated in 2D conditions nor in non-transformed cells [34]. We found that DGKα silencing or inhibition prevented cancer cell growth in 3D culture as well as tumor growth *in vivo*. Analysis of DGKα expression and Src activation under 3D conditions provided a mechanistic link by showing that DGKα expression has a positive regulatory function in Src activation. DGKα contribution to this central node helps to explain why targeting of DGKα specifically alters tumor viability, with no effect on untransformed cell survival. We identified DGKα as a Src-interacting partner that is necessary to sustain Src signaling and whose targeting improved the effect of Src and PI3K/Akt inhibitors. Our findings indicate that DGKα might be an effective target for anti-cancer therapies, alone or in combination with other widely used inhibitors.

## RESULTS

### DGKα is necessary for cancer cell growth in 3D culture

DGKα has been studied in several cancer models, where experimental evidence has shown that it mediates various aspects of cancer cell progression. No studies have addressed the contribution of this isoform to colon cancer so we selected the SW480 cell line, which was derived from a primary colorectal tumor and shows characteristics of highly transformed cells. The contribution of DGKα to the tumoral properties of SW480 cells was addressed by reducing its expression using interferent RNA. Previous validated DGKα target sequence [35, 36] efficiently reduced the expression of the protein in SW480 cells (Fig. S1A). Expression of DGKζ, an isoform that complements some DGK functions in T lymphocytes, was not altered in DGKα silenced cells (Fig. S1A). To evaluate the contribution of DGKα during long-term culture, the targeting sequence was cloned as shRNA in the pSuperRetro vector; this was then used to generate cells with stable reduction of DGKα levels (Fig. S1B).

**Figure 1:**
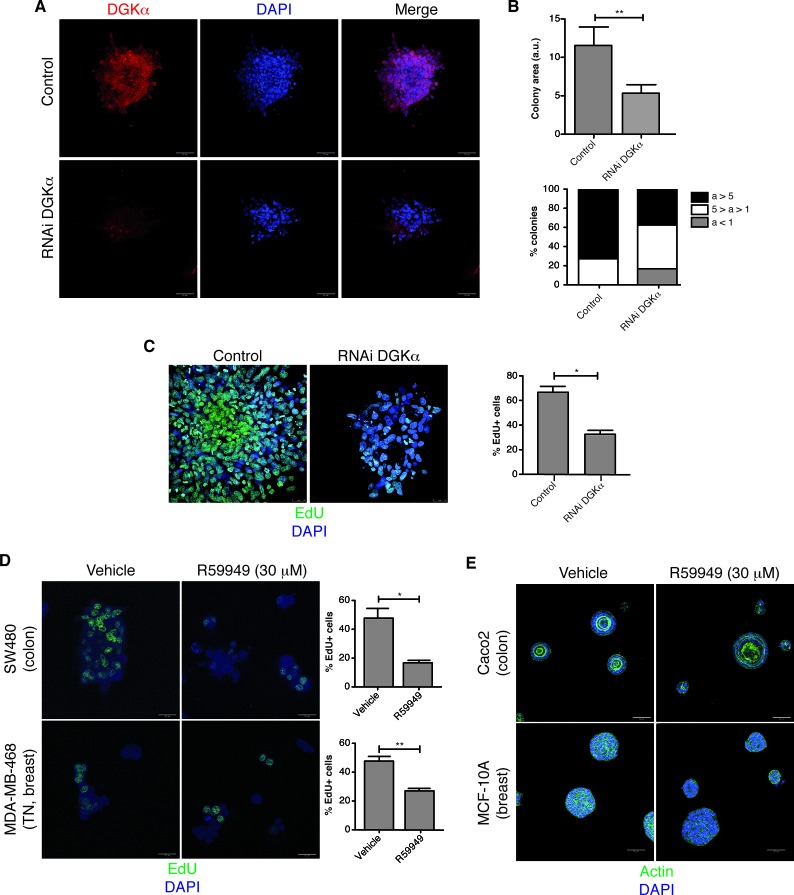
DGKα is necessary for cancer cell growth in 3D conditions (A) Stably control or DGKα-depleted SW480 cells were seeded on matrigel and cultured for 6 days. Cells were stained for DGKα and with DAPI. (B) Colony area (a) from control and DGKα-depleted cells was determined using the ImageJ software, and expressed in arbitrary units (a.u.). The mean ± SEM of the area of each group is shown (top). The area value was classified in 3 categories (a>5, 5>a>1, a<1 a. u.), the area distribution is showed for each group (bottom). n=50 colonies in each case. (C) Cell nuclei were stained with DAPI and proliferation inferred by staining with EdU. % EdU positive cells (mean ± SEM) in the total of DAPI stained cells was determined in several fields of at least 3 different experiments. (D) SW480 and MDA-MB-468 cells were seeded on matrigel (4 days) and R59949-treated (48 h). Cells were stained and analyzed as in C. (E) Caco-2 and MCF-10A cells were seeded on matrigel (4 days), R59949-treated (48 h), and stained for actin and with DAPI. A representative field is shown (*n* ≥3 independent experiments). A, C, D, bar = 50 μm; B, bar = 25 μm.

The effect of DGKα downmodulation on long-term growth of SW480 cells was evaluated in matrigel 3D culture and on plastic, 2D culture conditions. In 3D culture, SW480 cells showed a disorganized structure with poor cell-cell contacts, characteristic of highly transformed tumors [37]. Reduced DGKα expression in 3D cultures of the stably downregulated SW480 cells was corroborated by immunofluorescence (Fig. 1A). When compared to their control counterpart, cells with reduced DGKα levels formed smaller colonies (Fig. 1B). DGKα attenuation impaired cell proliferation, as revealed the reduced number of cells incorporating EdU (5-ethynyl-2 deoxyuridine), a thymidine analogue that measures DNA synthesis (Fig 1C).

Reduction of DGKα protein levels did not significantly affect cell growth in 2D; these cells formed colonies at the same extent that control cells (Fig. S2A). The effect of reduced DGKα expression on cell growth in either 2D or 3D conditions was compared by measuring cell viability with a tetrazolium reduction based assay (MTS). Simultaneous MTS measurements confirmed that DGKα silencing affected the viability of SW480 cells only when in 3D (Fig. S2B). These observations indicate that DGKα, whereas dispensable for 2D cell growth, is central for sustaining cancer cell growth in a 3D context.

Cancer cell growth in 3D induces tumorigenic traits that cells display *in vivo* and are not recapitulated in 2D culture. The contribution of DGKα to SW480 growth in 3D suggests that this enzyme could be of interest for cancer therapy. To study the potential of this pathway as a target for pharmacological intervention, we next compared the effect of diminishing DGKα protein levels with that produced by a pharmacological inhibitor. We selected the DGK inhibitor II (R59949) that binds to and blocks DGKα catalytic functions [38]. R59949 is reported to be more efficient that the other DGK inhibitor (R59022) in blocking the Ca^2+^-dependent type I DGK isoforms *in vitro*, mainly DGKα. R59949 inhibits purified DGKα in the low micromolar range [38], but it is highly lipophilic and tends to be sequestered by serum lipids when added to cell cultures [39]. By this reason the drug is used in serum-free culture conditions or, if serum is present, higher concentrations must be used. In this case R59949 may also inhibit other DGK isoforms [40]. 3D assays required long-term cell culture in serum-supplemented media so we first assessed the expression of additional DGK isoforms in SW480 cells.

Data from our related studies indicated that in addition to DGKα, SW480 cells also expressed DGKζ (Fig. S1B). Due to the lack of efficient commercial anti-DGK antibodies, we tested the expression of the remaining DGK isoforms by RT-PCR. This analysis indicated that SW480 cells also expressed type II DGKδ and DGKη, and type III DGKε; but not type IV DGKι or type V DGKθ (Fig. S3A). To discard that in our experimental conditions, R59949 could inhibit not only DGKα, but also some of the other expressed DGK, we determined the efficiency and selectivity of R59949 by comparing its effects over total DGK activity in control and DGKα silenced cells. We have previously used 30 μM of the inhibitor to demonstrate the DGKα-mediated control of hypoxia-inducible factor-1α; this same dose has been reported by other groups to confirm DGK function [41-43]. We thus cultured SW480 cells for 24 h in complete medium in the presence of R59949 at 15 and 30 μM. The lower dose had a minimum effect over total DGK activity, whereas 30 μM R59949 diminished DGK activity by near 25%. A similar reduction was observed in cells with DGKα silencing, and in this case R59949 addition did not further affect activity (Fig. S3B).

We next investigated if the R59949-resistant DGK activity in SW480 cells could be ascribed to the DGKζ isoform that, as we have previously shown, has a prominent role in controlling total DGK activity in T cells [36, 44]. We found that DGKζ contribution was also important in SW480 cells, since its targeting led to a severe reduction in total DGK activity (Fig. S3B). R59949 addition to DGKζ-silenced cells fully abolished DGK activity, confirming the drug sensitivity of the remaining isoforms, most probably DGKα, in these cells. This agrees with previous reports indicating the high sensitivity of DGKα to R59949 [38, 40], and further indicates that this isoform is the main target of R59949 in these cells.

The effect of pharmacological DGK inhibition was next assessed using SW480 cells growing in 3D. As for DGKα depletion, R59949 treatment of matrigel-plated SW480 cells reduced colony formation (Fig 1D, upper panel). Results were similar for the breast cancer cell line MDA-MB-468, which showed spheroid-like growth with grape-like morphology [37] (Fig. 1D, lower panels). Quantification of EdU incorporation confirmed that, as shown for DGKα silenced cells, R59949 treatment diminished proliferation in the two cell lines (Fig. 1D).

Different from these two highly transformed cells, some cell lines form well-organized acinar-like structures that resemble normal epithelia when cultured under 3D conditions [37]. To be appropriate for clinical use, DGKα inhibition should have little or no effect on untransformed cell growth. We next tested the effect of DGKα inhibition on the Caco-2 cell line that, although it is classified as colon cancer, forms acinar-like structures in matrigel culture and is widely used to study colon morphogenesis [45]. R59949-induced DGKα inhibition did not affect the acinar-like morphology of these cells that grew at the same extent that vehicle-treated cells (Fig. 1E, S2C). R59949 had no effect on the polarized growth of MCF-10A cells, a breast-derived cell line that forms organized structures in 3D culture [46], Fig.1E, S2C). These data indicate that the disorganized growth characteristics of transformed cells require DGKα.

### DGKα depletion impairs tumor growth *in vivo*

**Figure 2:**
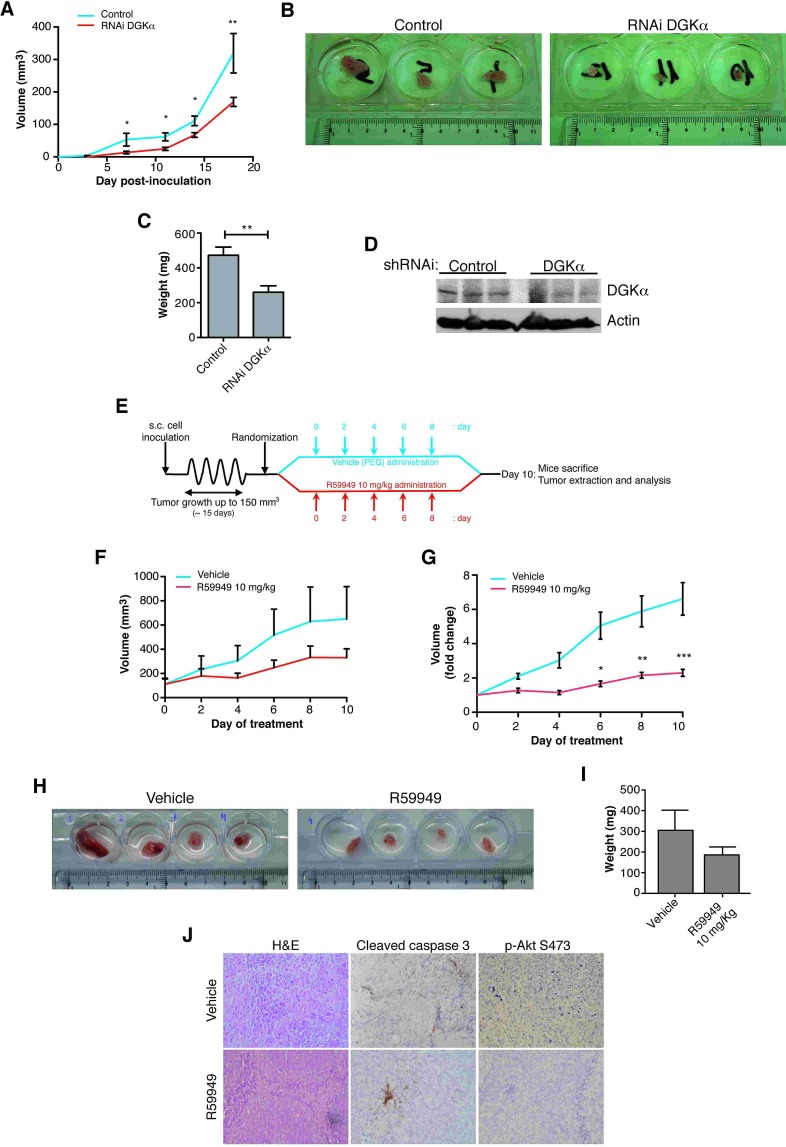
DGKα depletion impairs tumor growth *in vivo* Stably infected control or DGKα-depleted SW480 cells (1.5 × 10^6^) were injected s.c. into immunosuppressed mice (*n* = 6 mice/group). (A) Tumor volume was recorded every 48 h. The mean ± SEM of the volume of each mice group is shown. (B) After sacrifice, tumors were resected. Tumors of DGKα-depleted cells showed a minor size that those from control cells. (C) Tumor weighed (mean ± SEM). (D) Tumors were lysed in RIPA buffer using a tissue homogenizer and DGKα expression was determined by western blot. Actin was used as loading control (E) Diagram showing the schedule for testing the potential of targeting DGKα in a xenograft assay. SW480 cells (10^6^) were injected s.c. into immunosuppressed mice. When tumor volume reached ~150 mm^3^, mice were divided in two random groups (*n* = 6 mice/group) and treated s.c. with vehicle or R59949 every 48 h for 10 days. Tumor volume was recorded every 48 h. Mice were sacrificed at day 10 of the treatment. The volume mean (F) and the *x*-fold change (G) mean ± SEM in each case are shown. (H, I) After sacrifice the tumors were resected and weighed (mean ± SEM). (J) Tumors were fixed and stained with hematoxylin-eosin and for cleaved caspase 3 and AKT phosphorylated at S473. Each experiment was repeated twice.

Silencing of DGKα had no effect on cancer cell growth in 2D culture, at difference from that observed when cells were cultured in 3D. This characteristic suggests that DGKα sustains three-dimensional configuration *in vitro*, and thus possibly *in vivo*. We next examined the contribution of DGKα to maintain 3D growth *in vivo*, using DGKα silenced SW480 colon cancer cells in xenograft assay models. Mice injected with stably silenced DGKα SW480 cells developed tumors, but they appeared later and grew less compared to control cells (Fig. 2A). Tumors generated upon injection of DGKα silenced cells were smaller and weighted ~50% less than those of control cells (Fig. 2B, C). Western blot analysis of tumor lysates confirmed that DGKα silencing was maintained along the experiment (Fig. 2D).

To evaluate the *in vivo* potential of pharmacological DGKα targeting, we also determined the effect of the R59949 inhibitor on SW480 cell xenografts. Our group has reported that DGK inhibitors and rapamycin have similar effects over cell proliferation [47]. We thus chose a dose of 10 mg/kg of the inhibitor; similar to that used for rapamycin in xenograft assays [48]. In a pilot study, intraperitoneal (i.p.) administration of the corresponding volume of DMSO or R59949 in DMSO resulted highly toxic for the mice. We then selected an alternate vehicle for R59949 administration, using the drug in emulsion with 50% of PEG3000 in PBS. Again i.p. administration of PEG either alone or with R59949 was toxic at long term (50% mice from each group died following the second dose of treatment). Finally, R59949 in emulsion with PEG3000 was subcutaneously administrated. Mice were injected with SW480 cells, tumors were let to grow and R59949 was first injected when the tumors reached a volume of approximate 150 mm^3^. After the initial injection, the inhibitor was continuously injected every 48 h during 10 days, when tumors in control animals reached the maximal authorized size (Fig. 2E). This treatment was not toxic for the animals, as assessed by the lack of differences in the weight of vehicle and inhibitor treated mice (Fig. S4). R59949-treatment resulted in marked reduction of tumor growth (Fig. 2F). The impairment in tumor growth was statistically significant when the volume of each individual tumor was normalized to that measured prior inhibitor injection (Fig. 2G). Although R59949 treatment did not result in tumor regression, tumors excised from R59949-treated mice were smaller and weighted less than those obtained from vehicle-treated mice (Fig. 2H, I). Immunohistochemical analysis revealed reduced Akt phosphorylation and increased cleaved caspase 3 staining in tumors from R59949-treated mice (Fig. 2J). This analysis strongly suggests that pharmacological DGKα inhibition correlated with impairment of survival pathways and increased apoptosis.

### DGKα promotes Src activation

**Figure 3:**
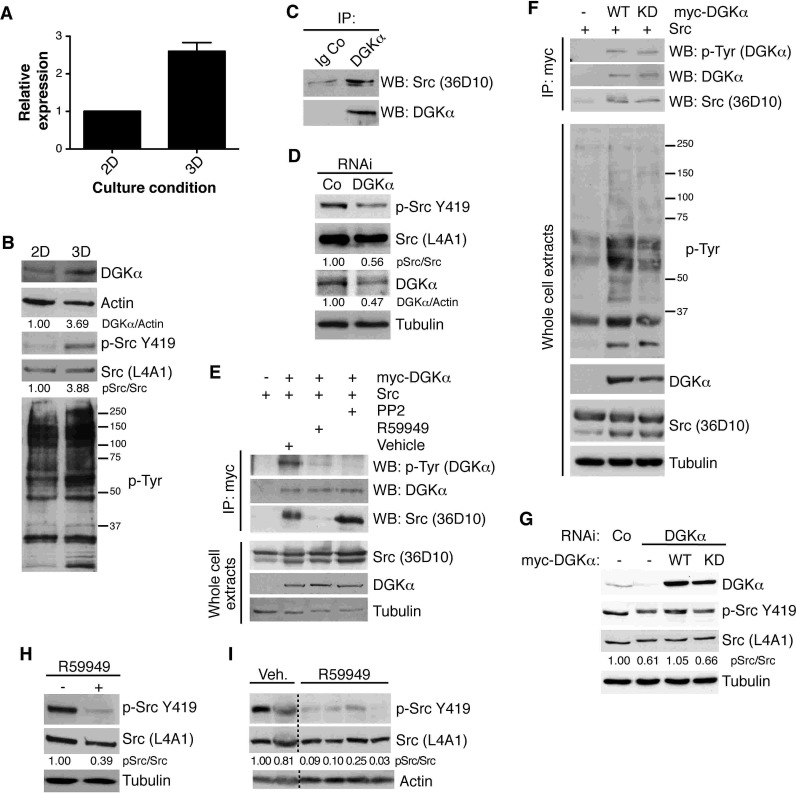
DGKα levels increase in 3D cell cultures and regulate Src signaling networks SW480 cells were plated at subconfluence (2 days) or on matrigel (6 days). (A) RNA was extracted and *DGKα* analyzed by qPCR. Expression was normalized to that of GAPDH. Mean SEM, *n* = 3. (B) DGKα levels, active Src and phosphotyrosine profiles were analyzed by western blot. Total Src and actin were used as loading controls. (C) DGKα immunoprecipitates of SW480 cells were probed for Src association. An unrelated antibody was used as a control. (D) Active Src was evaluated in SW480 cells in exponential growth with reduced DGKα levels. Total Src and tubulin were used as loading controls. (E) Chicken Src and human Myc-DGKα were transiently transfected in HEK-293 cells. After 24 h, cells were treated for 6 h with R59949 (20 μM) or PP2 (10 μM), and Myc-DGKα immunoprecipitated to analyze its phosphorylation and association with Src. (F) As in E, but wild type (WT) or kinase-dead (KD) Myc-DGKα were transfected. Phosphotyrosine profiles were analyzed by western blot. (G) SW480 cells were transiently transfected with control, scramble RNAi or targeting DGKα. 72 h post-transfection, the indicated Myc-tagged DGKα constructions were transfected. DGKα levels and active Src were analyzed by western blot. Total Src and actin were used as loading controls. (H, I) Active Src was evaluated in SW480 cells in exponential growth treated with 30 μM R59949 (H), or in tumors from R59949-treated mice (I). Total Src, tubulin and actin were used as loading controls. Representative blots are shown (*n* ≥3 independent experiments, except *n*=2 in I).

Our results suggested that DGKα participates in a central node that promotes cancer cell growth in a 3D context. Culture on matrigel matrices closely mimics the tumor microenvironment and reproduces the cell reprogramming that occurs *in vivo*. The *DGKα* promoter contains several regulatory regions that, as shown in T lymphocytes, modulate transcriptional expression of this enzyme along differentiation stages [14]. We thus investigated if DGKα expression varied as a result of 3D culture conditions. DGKα mRNA levels were ~3-fold higher in 3D-cultured SW480 cells compared to 2D culture (Fig. 3A), suggesting 3D-mediated upregulation of DGKα gene expression. We found correlation between increases in DGKα mRNA and in the total amount of DGKα protein (Fig. 3B). Higher DGKα protein levels were also observed for breast tumor-derived cell lines in 3D cultures suggesting a direct correlation with 3D imposed restrains (Fig. S5). An increase in tyrosine signaling networks, specifically those of the Src family, is a characteristic of colon and breast cancers [49, 50], and is critical to sustain disorganized growth under 3D conditions [51]. SW480 cells cultured in 3D conditions also showed greater Src phosphorylation at Y419, an autophosphorylation site that correlates with optimal Src activity. The increase in Y419 phosphorylation was accompanied by an overall increase in the phosphotyrosine profile (Fig. 3B).

DGKα is known to be a SFK-regulated target in T cells, as well as in cells of endothelial and epithelial origin. A recombinant GST-DGKα version interacts with and is phosphorylated by recombinant Src [18], and DGKα membrane recruitment and/or activity are induced by SFK-phosphorylation [16, 17]. Other Src effectors, through their association with Src, facilitate an open conformation of the kinase and thus its activation [52]. As increased Src activation in 3D conditions coincided with higher DGKα levels, with no obvious change in Src protein expression, we explored a possible correlation between DGKα upregulation and Src activation. Immunoprecipitation assays showed that endogenous DGKα associated to Src in SW480 cells (Fig. 3C). In these cells, DGKα silencing correlated with a notable reduction in Src Y419 phosphorylation, indicating Src inactivation (Fig. 3D). These observations suggest a mutual regulation between Src and DGKα where, as described for other SFK targets, DGKα interaction with Src facilitates Src activation.

To study in more detail the requirements for Src and DGKα interaction, we overexpressed both proteins in HEK293 cells, which do not express DGKα. DGKα was overexpressed as a Myc-tagged protein, to facilitate its immunoprecipitation. As observed for the endogenous proteins, the ectopically expressed DGKα interacted with Src and phosphotyrosine analysis confirmed DGKα phosphorylation (Fig. 3E). Treatment with the Src inhibitor PP2 did not disrupt DGKα−Src interaction, although it abolished tyrosine phosphorylation of DGKα. In contrast, treatment with the DGK inhibitor impaired DGKα−Src interaction (Fig. 3E).

To determine if the lack of DGKα−Src interaction was a direct consequence of impaired DGK activity, we examined the interaction of Src with a kinase-deficient DGKα mutant. DGKα, rendered inactive by mutation of the conserved, ATP-binding GGDG sequence [53], interacted with Src and was tyrosine phosphorylated (Fig. 3F). This indicates that DGKα enzymatic activity was dispensable for its interaction with Src. It also suggests that R59949-mediated complex disruption is probably the result of sterically hindrance following inhibitor binding to the DGKα catalytic domain. Total phosphotyrosine profile analysis, however, suggested increased Src activity in cells expressing the active DGKα construct (Fig. 3F), suggesting some contribution of DGKα activity for full Src activation.

Determination of Src activation in HEK293 cells was limited by the high level of ectopically expressed Src as well as by the expression of additional SFK members (see additional bands in HEK293 lysates). We thus reconstituted DGKα-silenced SW480 cells with wild type and kinase-dead DGKα versions (Fig. 3G). In these conditions, full Src phosphorylation was only reconstituted by wild type DGKα but not by the kinase-dead mutant. This suggests that the enzymatic activity of DGKα is required to achieve complete activation of endogenous Src. R59949 treatment mimicked the effect of DGKα attenuation and resulted in a strong reduction of Src Y419 phosphorylation (Fig. 3H). Finally, the analysis of Src activation in tumor lysates from vehicle- and R59949-treated mice bearing SW480 induced tumors, confirmed that treatment with R59949 *in vivo* also diminished Src activation (Fig. 3I).

### DGKα silencing increases cancer cell sensitivity to Src inhibition

**Figure 4:**
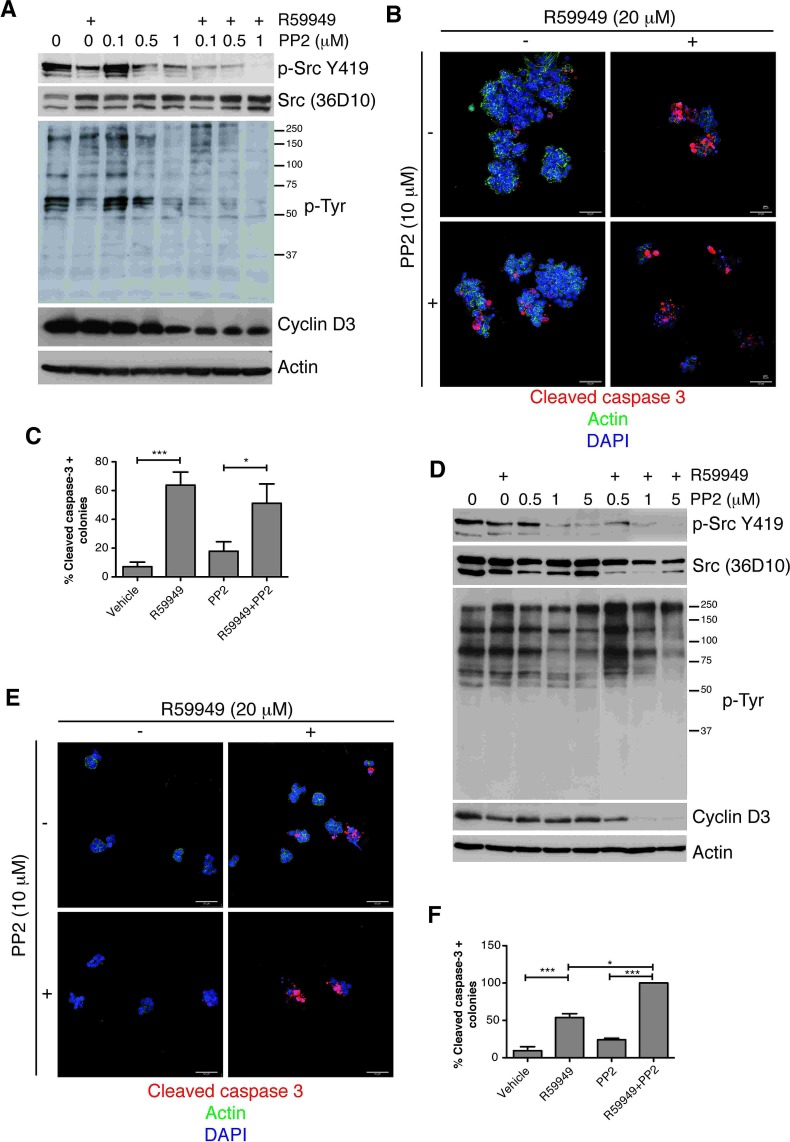
Blocking DGKα augments the sensitivity of cancer cells to Src inhibition (A) SW480 cells cultured on 2D conditions were treated with the DGK inhibitor (20 μM), the indicated doses of PP2, or both (24 h). Active Src, phosphotyrosine profiles and cyclin D3 levels were analyzed by western blot. Actin was used as loading control. (B) SW480 cells were seeded on matrigel (4 days), and treated with inhibitors (48 h). Cells were fixed and apoptosis evaluated as cleaved caspase 3 staining. Actin and DAPI staining were also performed. (C) The number of caspase positive colonies was determined by examining several fields. The mean ± SEM of the percentage obtained for each group in different experiments is shown. (D-F) The PTEN-negative cell line MDA-MB-468 was treated with indicated drugs and analyzed as in A, B or C, respectively. Representative blots and fields are shown (*n* ≥3 independent experiments). Bar = 50 μm.

Despite the importance of Src in malignant transformation, monotherapy using Src inhibitors in clinical trials has had only modest results [54]. Aspects of the 3D tumor environment may contribute to limit drug effectiveness including the spatial activation of Src and its regulation by interacting partners. The previous experiments suggested that DGK inhibitor treatment impaired DGKα interaction with Src, so we hypothesized that disruption of DGKα-Src complex could not only diminish Src activation, but also enhance its sensitivity to pharmacological inhibitors. Treatment of SW480 cells with the Src inhibitor PP2 diminished Src phosphorylation similar to that observed when treating cells with the DGK inhibitor. The dose of PP2 required to impair Src phosphorylation was reduced when SW480 cells were co-treated with the DGK inhibitor (Fig. 4A). Decreased Src phosphorylation correlated with diminished total tyrosine phosphorylation and reduced expression of Cyclin D3, a cell cycle promoter that is upregulated in many tumor cells [55].

The effect of drug combination was also evaluated in 3D cultures using cleavage caspase 3 as an indicator of apoptotic cell death. In these experiments PP2 was assayed at 10 μM, a concentration reported when using 3D conditions to avoid diffusion and lack of effectiveness due to matrix structure [51]. PP2 treatment at this concentration partially reduced 3D growth and promoted apoptosis of SW480 cells (Fig. 4B, C). R59949 promoted apoptosis to higher extent, independently of PP2 addition (Fig. 4C). Simultaneous addition of the two inhibitors, however, affected colony integrity as denoted by the decrease in the percentage of intact colonies (Fig. 4B and S6A).

Resistance to Src inhibition has been also attributed to the presence of additional oncogenic mutations. Lack of the PTEN phosphatase, a frequent mutation in breast tumors, enhances Akt activation and phosphotyrosine signaling, which diminishes the efficacy of drugs that block RTK or SFK [56]. As for SW480 cells, simultaneous treatment of the PTEN negative MDA-MB-468 cells with R59949 and PP2 reduced Src phosphorylation and Cyclin D3 levels (Fig. 4D). Different from that observed in SW480 cells, phosphotyrosine profiles did not decrease accordingly and even some proteins of high molecular weight showed increased phosphorylation (Fig. 4D). PP2 treatment promoted apoptosis of MDA-MB-468 cells grown in matrigel, although, as for SW480 cells, cell death increased if cells were treated with R59949 (Fig. 4E, F). Combination of PP2 with R59949 significantly increased apoptosis compared to individual treatments (Fig. 4F), and also increased the number of colonies with a disintegrated integrity pattern (Fig. S6B).

Contrary to their previous effects, either individual or combined addition of R59949 and PP2 did not affect spheroid formation when added to 3D cultures of Caco-2 or MCF-10A cells (Fig. S7). Our data concur with previous studies showing the synergistic effect of PP2 with other inhibitors acting on the Src pathway in cancer cells, as has been recently described for PKCα inhibitors in breast cancer [57]. These findings strongly suggest that DGKα targeting could render cancer cells sensitive to Src inhibitors.

### DGKα participates in the adaptive response of cancer cells to PI3K/Akt inhibition

*DGKα* gene expression is strictly controlled by FoxO factors; they promote transcriptional activity by binding to the *DGKα* distal region [14]. Akt-mediated phosphorylation of FoxO factors leads to their nuclear exit, which blocks their transactivatory function [58]. DGKα function thus lies downstream of the PI3K/Akt pathway and its function is expected be coupled to the cell differentiation programs controlled by FoxO family. Accordingly, DGKα expression in cytotoxic T populations is repressed by activation of the PI3K/Akt pathway [14]. FoxO factors also control expression of several receptor and non-receptor tyrosine kinases. Noteworthy, cancer cells rely on this mechanism to trigger tyrosine kinase signaling when PI3K or Akt inhibitors are used, which makes them resistant to the deleterious effects of these drugs [59, 60].

**Figure 5:**
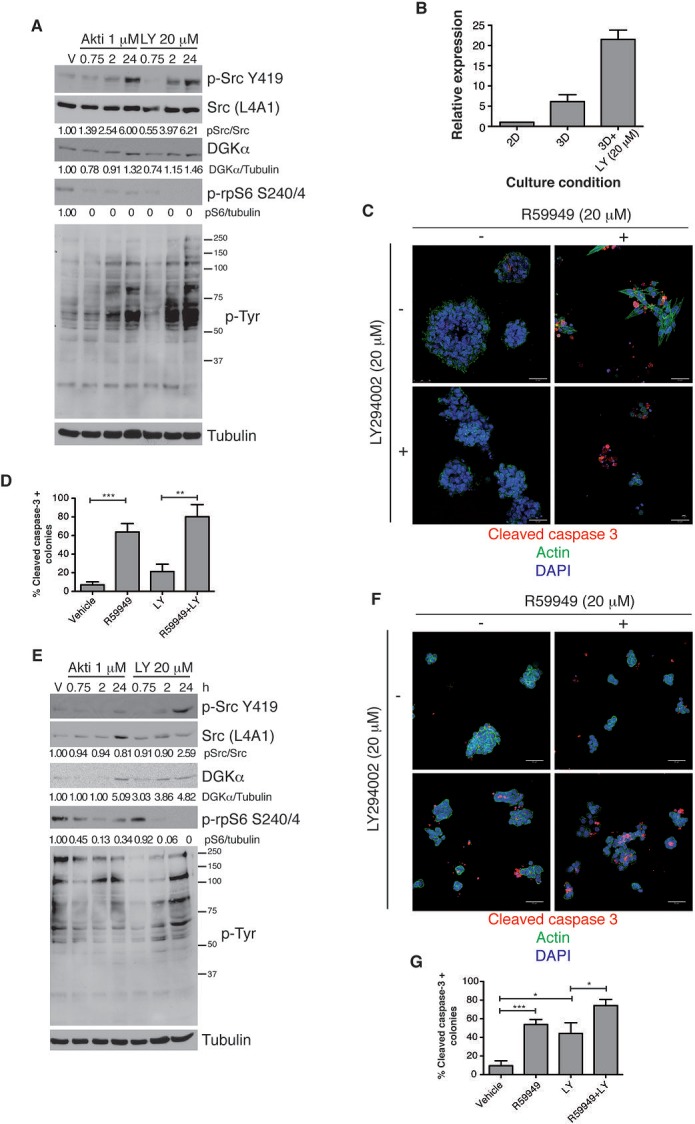
DGKα expression increases after PI3K inhibition (A) SW480 cells were treated with Akt or PI3K inhibitors for the times indicated. Active Src, phosphotyrosine profiles and DGKα levels were analyzed by western blot. Phosphorylation of rpS6 was used as control of the inhibition. Tubulin was used as loading control. pSrc was normalized to Src expression. DGKα and prpS6 levels were normalized to those of tubulin. (B) SW480 cells were cultured in 2D or in 3D. LY294002 was added to the 3D culture (48 h). RNA was extracted and *DGKα* analyzed by qPCR. Expression was normalized to that of GAPDH. Mean ± SEM, *n* = 3. (C) SW480 cells were plated on matrigel and treated with R59949, LY294002, or both (48 h). Cells were fixed and stained for actin and caspase 3 and with DAPI. (D) The number of caspase positive colonies was determined by examining several fields from different experiments. The mean ± SEM of the percentage obtained for each group in different experiments is shown. (E-G) MDA-MB-468 cells were treated as in A, C or D. In A and E representative blots are shown (*n* =3 independent experiments). In C and F representative fields are shown (*n* ≥3 independent experiments). Bar = 50 μm.

We tested whether PI3K/Akt inhibition in SW480 colon cancer cells triggered activation of Src and tyrosine signaling, and if this correlated with transcriptional upregulation of DGKα. PI3K or Akt-inhibited cell cultures of SW480 cell showed increases in Src activation and in total phosphotyrosine profiles (Fig. 5A). DGKα protein levels increased after 24 h of treatment, a time in which Src activation reached the maximum grade (Fig. 5A). Since in 3D culture conditions the expression of DGKα was higher than in 2D, we next determined its levels in this condition after PI3K inhibition. qPCR analysis of *DGKα* mRNA levels in PI3K/Akt-inhibited 3D cell cultures of SW480 cells showed a 3- to 5-fold increase in *DGKα* expression relative to controls (Fig. 5B). By sustaining Src activation, increased DGKα levels might limit the deleterious effect of PI3K/Akt inhibitors on tumor survival. In agreement, combined inhibition of DGK and PI3K diminished cell growth and increased apoptosis more effectively than PI3K inhibition alone (Fig. 5C, D). The combined treatment also led to higher disruption of colony integrity compared to any of the single treatments (Fig. S6A).

Increases in Src activation and DGKα protein levels were also observed after PI3K/Akt inhibition in MDA-MB-468 cells (Fig. 5E). In these cells the effects were stronger when the PI3K inhibitor was used instead of the Akt inhibitor, probably due to the lack of PTEN. Changes on Src activation where not reflected in these cells in the total phosphotyrosine profile, differently from that observed in SW480 cells. In 3D culture conditions, PI3K inhibition promoted apoptosis at similar extent that R59949 (Fig. 5F, G), although the combined inhibition of DGK and PI3K increased the number of colonies with disintegrated structure (Fig. S6B). Inhibitors treatment did not affect spheroid formation by Caco-2 or MCF-10A cells when added either alone or in combination (Fig. S7).

Together these data suggest that the crosstalk between the PI3K/Akt/FoxO and Src pathways via DGKα provides cancer cells with a mechanism to adapt and subvert the deleterious effects of PI3K/Akt inhibition. DGKα targeting might therefore represent a useful and non-explored approach to combine with and prevent resistance to anti-cancer therapies aimed to inhibit the PI3K/AKT axis.

## DISCUSSION

Improvement of anticancer therapies requires reliable identification of targets with a clear role in cancer cell adaptation to the cues of the tumor microenvironment. Three-dimensional cell culture constitutes a valuable tool for target identification, since it resembles the conditions that cells might encounter *in vivo* [61]. Compared to 2D, 3D-cultured cells show patent differences not only in morphology, but also in the expression of various receptors and oncogenes, and in the response to drugs [62]. Here we identify DGKα upregulation as part of the reprogramming that occurs when breast and colon cancer cells grow in a 3D microenvironment. In addition, we demonstrate that DGKα silencing or its pharmacological targeting limits cell growth only when cells are 3D-cultured or xenografted in immune-compromised mice, but not during 2D cultures. Our data reinforce previous reports supporting a positive function of DGKα in cancer, and also provide a mechanistic link by showing that DGKα contributes to Src activation. These findings identify DGKα as a component of a positive feedback loop that contributes to Src-regulated functions and as a potential target for the development of anti-tumor therapies

Several reports indicate that DGKα sustains survival of cancer cells, including lymphoma, melanoma and hepatoma [2-4]. DGKα phosphorylation by Src correlates with the activation of its lipid kinase activity [17, 18], leading to PA generation. In this study we demonstrate that DGKα, in addition to its previous identification as a Src target, may also contribute to enhance Src functions. The mechanisms that control Src hyperactivation in cancer remain unclear [30] although it is becoming apparent that oncogenic Src functions are largely dictated by protein interactions that facilitate/restrict its activation. These modulators include upstream RTK, kinases and phosphatases that regulate Src conformation, as well as substrates that unclamp closed/inactive Src and lead to an open/active state [20, 63-65]. Additionally, Src activity is conditioned by its spatial localization or compartmentalization in distinct membrane microdomains [66, 67]. The unique features of DGKα might allow it to modulate Src at several levels, and thus contribute to its oncogenic properties.

The correlation between DGKα expression and Src autophosphorylation at Y419 correlates with the well-established mechanism of Src activation as a result of substrate interaction. Src modular structure facilitates activation by its substrates through a sequential mechanism: Src SH3 domain mediates the recognition of targets that contain proline-rich sequences. Subsequent phosphorylation leads to exposure of an additional Src docking site, generally a SH2 binding motif, promoting an open/active conformation of the kinase [21, 68]. DGKα does not have a consensus SH3 interaction motif, but its C-terminal region contains a proline stretch that interacts with Src *in vitro*. This interaction drives subsequent phosphorylation of DGKα at Y335 providing additional anchorage to Src SH2 motif [18]. Our studies suggest that DGKα, like FAK and CAS, might belong to the family of Src substrates with capacity to promote Src activation.

The capacity of DGKα to trigger Src activation by means of interaction would be independent of its function as a PA producer. In agreement, we show that a kinase inactive DGKα was still capable to bind Src and become phosphorylated. Nonetheless, reconstitution experiments strongly suggest that DGKα activity contributes to Src activation. In this regard, recent studies have identified motifs for binding of acid lipids, including PA, in the Src unique and SH3 domains [69]. PA binding to Src would thus represent an additional layer of regulation to modify Src conformation and activity. In addition to PA mediated Src regulation, others have shown that DGKα-generated PA contributes to the survival and invasive properties of cancer cells by several mechanisms. DGKα-derived PA promotes mobilization and membrane tethering of RCP, a modulator of Rab protein trafficking, which is necessary for integrin recycling and cell migration in p53-mutant background [7]. DGKα-PA production activates phosphodiesterase activity, that controls cAMP levels and the expression of cAMP responsive genes critical for cancer cell survival including mTOR and HIF-1 [11].

Our results suggest a model in which DGKα interaction with Src would allow mutual regulation of both kinases in a complex, multilayered process that would enhance PA production and Src activation. A partial contribution of DGKα-catalytic activity may help to explain the stronger effects on 3D growth observed following treatment with a pharmacological inhibitor when compared to those obtained after DGKα silencing. Whereas both treatments decrease total DGK activity to a similar extent, we demonstrate that R59949 treatment also promotes disruption of the DGKα-Src complex, most probably as a consequence of steric interference. A partial DGKα depletion following genetic silencing (aprox. 60% of total enzyme) would be less effective than the inhibitor targeting complex formation.

This role for DGKα as a Src modulator helps to understand the stronger addiction for this lipid kinase of transformed cells when growing in 3D compared to non-transformed, acini forming cells. This is likely the consequence of the strict requirement that transformed cells have for Src-mediated regulation of growth and survival pathways in the context of matrix components. It also explains the apparent lack of effect when cells are grown in 2D cultures.

DGKα promotion of Src activation might also explain the higher DGKα levels found in hepatic tumors when compared to untransformed neighboring tissue [4]. DGKα is increased in 20% of hepatomas, where it is associated with high Ki67 levels and recurrence rate. In these studies, the authors suggest that DGKα promotes MEK/ERK activation by a Ras-independent mechanism. It is tempting to suggest a function for DGKα promoting Src signaling in this cancer type, since there is mounting evidence of a role for Src in hepatocellular carcinoma development [70].

Our studies define an important contribution of DGKα to Src mediated functions. The increased DGKα expression observed at distinct stages of cancer progression, that is, from initial to metastatic growth [13] correlates with the reported function of Src in metastasis and suggests DGKα upregulation in response to stress conditions, such as limited nutrient availability or chemotherapy treatments. In this latter case, our data demonstrate that DGKα expression, upregulated in response to PI3K/Akt inhibition, contributes to Src activation, and provide additional proof of DGKα as part of tumor escape mechanisms. In summary, DGKα upregulation in 3D context links the function of this enzyme to tumor survival and drug resistance mechanisms, and further supports development of new drug therapies based on this lipid kinase.

## MATERIALS AND METHODS

### Cell lines and culture

MCF-10A, HEK293 and SW480 cells were purchased from the ATCC. MDA-MB-468 was kindly donated by Dr. L. Planelles (CNB/CSIC) and Caco-2 by Dr. A. González (CNB/CSIC). SW480 cells were maintained in DMEM (Lonza) supplemented with 10% FBS (GBio) and 2 mM L-Gln (Gibco). Caco-2 cells were maintained with 1 mM sodium pyruvate (Gibco). MCF-10A cells were maintained in DMEM/F12 (Lonza, 1:1, v/v), 5% horse serum (Gibco), 2 mM L-Gln, insulin-transferrin-selenium (Gibco), 500 ng/ml hydrocortisone (Calbiochem) and 20 ng/mL epidermal growth factor (EGF, Upstate). MDA-MB-468 cells were cultured in Leibovitz's medium (L15, Lonza). All cell lines were maintained at 37ºC and 5% CO_2_ except MDA-MB-468, which was cultured without CO_2_ in L15 medium. Identity of the breast-derived cell lines was confirmed by STR genotyping (Genomics Facility, IIB/CSIC, Madrid, Spain).

### Antibodies and reagents

Antibodies to p-Src Y416, Src (clones 36D10 and L4A1), p-rpS6 S240/4, cyclin D3, cleaved-caspase 3 and myc-tag (clone 9B11) were purchased from Cell Signaling. Anti-DGKα was from Abnova, monoclonal anti-β-actin and -α-tubulin antibody from Sigma-Aldrich, and anti-GAPDH from Santa Cruz Biotechnology. Anti-phosphotyrosine (4G10) was from Upstate.

Leupeptin and aprotinin were from Roche, growth factor reduced-matrigel from BD Biosciences. R59949 (DGK inhibitor II; 3-{2-(4-[bis-(4-fluorophenyl) methylene]-1-piperidinyl)ethyl}-2,3-dihydro-2-thioxo-4(1*H*)quinazolinone), PP2, LY294002 and Akt Inhibitor VIII (Akti) were from Calbiochem. All other reagents were from Sigma-Aldrich.

### DGK silencing

DGK-targeting was performed using the human DGKα (nt 1153-1173) and DGKζ (nt 2290-2310) validated sequences [35, 36, 71]. For transient silencing the sequences were transfected as dsRNA (Ambion) using oligofectamine (Invitrogen). A scramble dsRNA was used as control (Ambion). Effective DGK depletion was achieved at 72-96 h post-transfection. After 96h the expression was recovered. For stable silencing, 64 pb double strand oligonucleotides, encompassing the corresponding interfering 21 nt sequence and a hairpin structure, were cloned in the pSuperRetro vector (Oligoengine). As control the pSuperRetro construct harboring the homolog specific DGKζ mouse sequence was used. This control did not reduce the levels of human DGKα or ζ. SW480 cells were infected using standard protocols with the pSuperRetro-cloned sequence-containing retroviruses. Cells with the retrovirus insertion were selected by culturing in media with G418 (800 μg/ml) until the control, non-infected cells died (~1 week). The pools were checked for DGK silencing, maintained in media with G418 (500 μg/ml) and used in the long-term experiments.

### Cell drug treatments

The different inhibitors were added to plated cells in complete media for the indicated time (24 to 48 h). For DGK inhibition the DGK inhibitor II (R59949) was used since this drug has been reported by our laboratory and others to be much more potent and specific that DGK inhibitor I [38, 47]. To avoid inactivation of R59949 by the serum present in the culture medium and/or the long times of treatment, inhibitor doses from 15 to 30 μM in DMSO were used in the experiments.

### DGK assays

DGK activity was measured using 1,2-dioleoyl (C18:1)-DAG as substrate. Lipid micelles containing the substrate were prepared by sonication of the lipid in the presence of PtdSer (10 min, room temperature (RT)); the final concentration of both lipids in the assay was 2 mM. Cells were lysed by sonication in Tris-HCl 50 mM pH 7.5, with protease and phosphatase inhibitors (20μM leupeptin, 1.5μM aprotinin, 1mM PMSF, 2mM sodium fluoride, 40mM β-glycerolphosphate and 1mM sodium orthovanadate). Cell lysates were centrifuged (12000 x g, 15 min, 4ºC), and 20 μg of the resulting supernatant mixed with lipid micelles containing the substrate. The reaction was initiated by addition of reaction mix (1 mM ATP, 10 mM MgCl_2_, 100 mM NaCl, 1 mM DTT and 10 μCi [γ^−32^P]ATP) and incubated (10 min, 25ºC) in a final volume of 50 μl, and was terminated by addition of 1 N HCl. Lipids were extracted after addition of CHCl_3_/MeOH (2:1 v/v). The organic layer was recovered, dried and applied to silica gel TLC plates, which were developed in a CHCl_3_/MeOH/4 M NH_4_OH solvent system (9:7:2 v/v/v), dried, and autoradiographed. A non-radioactive standard of the reaction product was used to identify the lipid of interest.

### Cell Proliferation

The effect of the distinct drugs over cell proliferation was determined by evaluating the cell viability using the Promega Cell Titer cell proliferaton assay and according to the manufacturer instructions. The cells were cultured in 96 well plates in either 2D or 3D conditions. Absorbance was measured at 492 nm in a Sunrise Tecan instrument.

### Expression vectors and transfection

Myc-DGKα cloned in pMT2 was a kind gift of Dr. A. Graziani (University of Piamonte Orientale, Italy). A kinase-dead version of the enzyme was generated, Gly 434 to Ala, using the Quick-Change mutagenesis kit. Oligonucleotides used were: DGKA-G434Aforw: 5′-TTGCTGTGTGGTGCAGACGGCACAGTA-3′, DGKA-G434Arev:5′-TACTGTGCCGCTTGCACCACACACCAA-3′. Nucleotides underlined encoded for the modified amino acid.

The plasmid encoding Src was generated by cloning chicken Src into pCDNA3. Transfections were performed with Lipofectamine LTX/Plus reagent (Invitrogen).

### Western blot

For lysates, cells were washed in cold PBS, and scraped into ice-cold lysis buffer (50 mM Hepes pH 7.4, 150 mM NaCl, 1% TritonX-100, 10% glycerol) with protease and phosphatase inhibitors (20 μM leupeptin, 1.5 μM aprotinin, 1 mM PMSF, 1 mM sodium orthovanadate, 40 mM β-glycerophosphate, 2 mM NaF). Clarified lysates were quantified with the Pierce 660 nm Protein Assay (Thermo Scientific), denatured in Laemmli buffer, and resolved by PAGE. Proteins were transferred to nitrocellulose membranes (Bio-Rad) and incubated with the indicated antibodies.

To prepare lysates from matrigel, cell cultures were scraped into PBS with 5 mM EDTA, protease and phosphatase inhibitors, and matrigel allowed to liquefy (30 min, 4ºC). Cells were centrifuged (2000 xg, 5 min, 4ºC), washed twice in PBS with 5 mM EDTA and processed as above.

### Immunoprecipitation

For protein-protein interaction analysis, cells were lysed (30 min, 4ºC) as above. Lysates (500 μg-1 mg) were incubated (overnight, 4ºC) with appropriate antibody, followed by 50 μl 50% protein G-Sepharose slurry (1 h, 4ºC). Immunoprecipitates were washed thrice in lysis buffer and once with 0.5 M LiCl. Immunoprecipitated proteins were resolved by SDS-PAGE and transferred to nitrocellulose membranes for Western blot analysis.

### 3D cell culture

3D cell culture on matrigel was performed as described [46]. Briefly, 300 μl matrigel was added to each well of a 6-well chamber (10 cm^2^ surface) and allowed to solidify (20 min, 37ºC). Cells were trypsinized, resuspended in assay medium (normal medium with 2.5% matrigel) and seeded (1.5 to 3 × 10^3^ cells/cm^2^). Assay medium was changed every 48 h, and cells allowed to grow up to 14 days. For inhibitor treatment, cells were allowed to grow for 4 days, and then treated for 2 additional days.

### Colony formation assays

Cells (5 × 10^2^/well) were seeded in 12-well plates and allowed to grow for 10 days. Colonies and foci were stained with 0.1% crystal violet solution in 20% methanol. After extensive washing, crystal violet was dissolved in 10% acetic acid, and absorbance measured at 620 nm in a Sunrise Tecan instrument.

### Immunofluorescence

Cells were plated on matrigel-coated (60-70 μl) 8-well chambers (Ibidi) and after treatment as indicated, were fixed in 4% paraformaldehyde (10 min, RT). Cells were permeabilized with 0.5% Triton X-100 (30 min), blocked with 10% goat serum, 0.05% TritonX-100 in TBS (30 min, 37ºC), and incubated with appropriate primary antibody in incubation buffer (1% goat serum in TBS; 3 h, 37ºC) in a humidified chamber. Secondary antibodies (goat anti-rabbit Alexa Fluor 488 or 594; both from Invitrogen) were added and incubated (1 h, RT). To assess proliferation, EdU (Click-iT EdU Cell Proliferation Assays, Life Technologies) was used as recommended. Finally, samples were incubated with DAPI (Invitrogen, 1:1000, 20 min, RT) and Alexa Fluor 488- or TRITC-conjugated phalloidin (Invitrogen, 1:1000, 20 min, RT), mounted in PBS:glycerol (1:1, v/v), and visualized under a Leica TCS SP5 confocal microscope.

### Real time PCR

RNA was extracted from cell cultures using Trizol reagent (Invitrogen). cDNA was synthesized from cell culture-extracted RNA (1 μg) after DNase (Invitrogen) treatment and retrotranscription with Random Primers (Applied Biosystems) and SuperScript II Reverse Transcriptase (Invitrogen). qPCR reactions were performed with the Power SYBR Green PCR Master Mix (Applied Biosystems) in 10 μl final volume. PCR conditions were as follows: 50ºC (10 min), 95ºC (2 min), 40 cycles at 95ºC (15 s) and 60ºC (1 min). Reactions were run in triplicate with the Applied Biosystems 7900HT system. Relative expression of each gene was calculated using the ΔΔCt method. GAPDH was used as control. Oligonucleotides used were: DGKAforw: 5′-CAATCACATCTGTGGGTGCGAGGA-3′, DGKArev: 5′-TTCCCGCCACTCTTAGGATTGAC-3′; GAPDHforw: 5′-ACAGCCTCAAGATCATCAGCAA-3′, GAPDHrev: 5′-ATGGCATGGACTGTGGTCATG-3′.

### Xenograft model

All mouse work was carried out in accordance with a protocol approved by the CNB/CSIC Ethics Committee for Animal Experimentation (CEEA-CNB, no. 090004). SW480-shRNA-control and -shRNA-DGKα cells (1.5 × 10^6^) were injected subcutaneously (s.c.) into the flank of female BALB/c SCID mice aged 6-8 weeks. Tumor growth was monitored every two days and volume estimated according to the formula: volume = (a^2^ × b)/2, where a = tumor width and b = tumor length in mm. At the end of the experiment (when control tumors reached 1 cm^3^, ~30 days), mice were sacrificed, tumors extracted and weighed.

To assay the effect of the DGK inhibitor R59949 on *in vivo* tumor growth, SW480 parental cells (10^6^) were injected s.c. into the flank of female mice as above. Pharmacological treatment was initiated when tumors reached ≥150 mm^3^ (~15 days). R59949 was administered s.c. (10 mg/kg) every 48 h for 10 days. The tumor volume and weight of each mouse was recorded along the treatment. Mice were sacrificed 48 h after the last dose. Tumors were weighed and then fixed overnight in paraformaldehyde, followed by dehydration in graded ethanol, and embedded in paraffin for immunohistochemical studies.

### Immunohistochemistry

Tissue sections were formalin-fixed and paraffin-embedded. Sections were prepared for antibody staining as described [72]. Briefly, following deparaffinization and hydration, antigen retrieval was performed in 12.5 mM sodium citrate pH 6.0 (30 min, 90ºC). Tissue sections were washed in TBSt (TBS, 0.1% Tween-20) and blocked with 10% goat serum (1 h, room temperature). Primary antibody was incubated (overnight, 4ºC), followed by incubation with HRP-coupled secondary antibody (Dako, 1:200, 2 h, room temperature). Staining was detected with the DAB Peroxidase Substrate Kit (Vector Laboratories), and slides were counterstained with hematoxylin (Fisher Scientific) and mounted in Permount (EMS).

### Statistical analyses

Gel bands were quantified with ImageJ software (NIH). Student's t-test was used to compare data. When variance was significantly different as analyzed with the F-test, Welch's correction was applied. When samples did not fit normality as tested with the Kolmogorov-Smirnov test, the Mann-Whitney test was used. Statistical analysis was done with GraphPad Prism 5 software. When the p-value <0.05, differences were considered significant (* p<0.05; ** p<0.01; *** p<0.001).

## SUPPLEMENTARY FIGURES


