# Supplementary material for: Diacylglycerol kinase α promotes 3D cancer cell growth and limits drug sensitivity through functional interaction with Src
Source: Oncotarget. 2014 Aug 12;5(20):9710–26. doi: 10.18632/oncotarget.2344 (PMC4259432; doi:10.18632/oncotarget.2344)
Supplement: Supplementary file 1 [file oncotarget-05-9710-s001.pdf]

# Diacylglycerol kinase α promotes 3D cancer cell growth and limits drug sensitivity through functional interaction with Src

## Supplementary Material

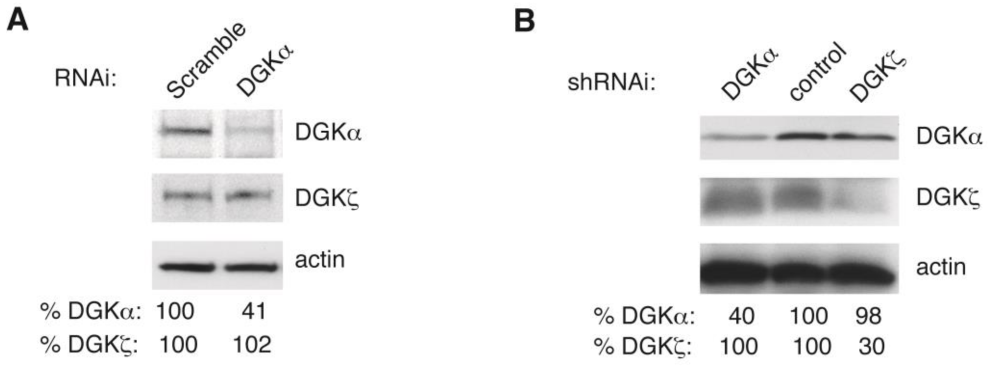


**Figure S1: DGKα downregulation in SW480 cells.** (A) For transient silencing SW480 cells were transfected with oligofectamine with either a scrambled (Ambion) control sequence or the siRNA against human DGKα. After 96 h post-transfection, cells were lysed and DGKα, levels analyzed by western blot. Tubuline was used as loading control. (B) Cells stably expressing shRNAi that targets the human DGKα, mouse DGKζ, or human DGKζ, were obtained by infection with pSuperRetro-cloned sequence-containing retroviruses and selected and maintained in culture with G418. Actin was used as loading control. Relative expression of each DGK is indicated below each blot.

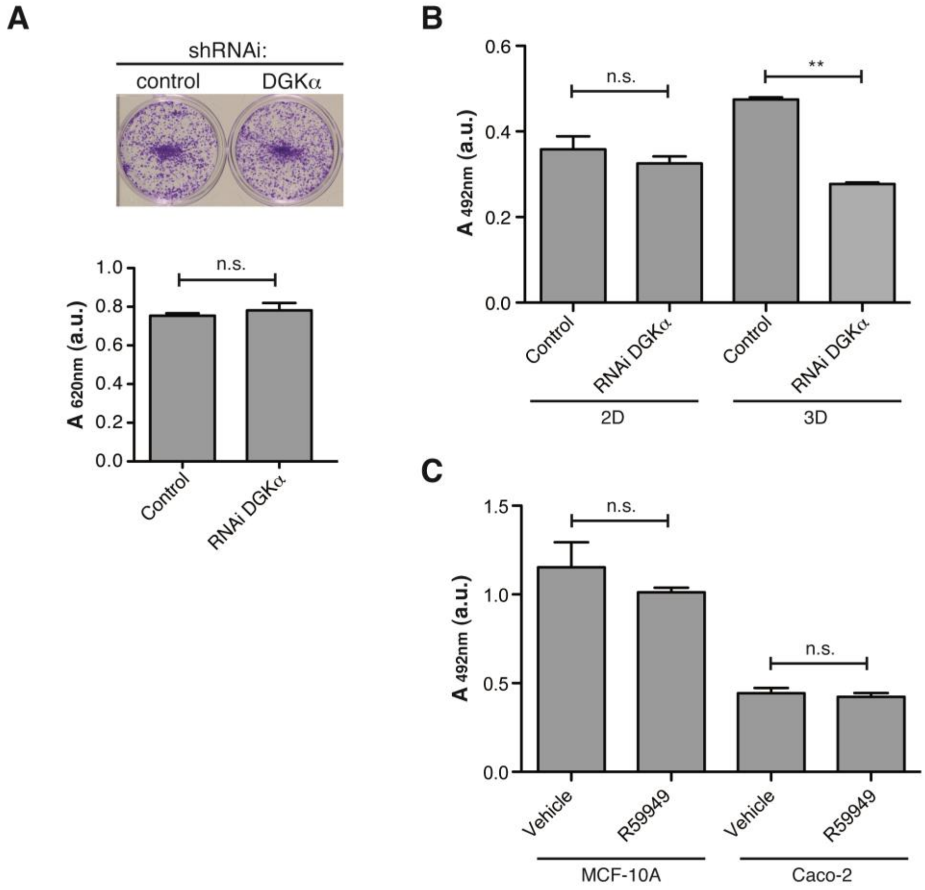


**Figure S2: DGKα silencing does not impair cell proliferation of SW480 cells in 2D culture conditions.** SW480 cells stably expressing shRNA control or against DGKα were cultured for several days under the conditions indicated. (A) Colony growth formation assays were performed culturing cells for 10 days on plastic in complete medium. Crystal violet staining was used to evaluate the colony number (top); cell growth was related to the amount of crystal violet incorporated by the culture. After staining the colorant was extracted and the absorbance at 620 nm determined. The mean ± SEM of absorbance is shown for triplicate samples. (B) Cells were cultured for several days on plastic or in 3D culture conditions and processed for MTS staining. The absorbance of the oxidized product was determined at 492 nm. The mean ± SEM of absorbance is shown for triplicate samples. (C) MCF-10A or Caco-2 cells were cultured in 3D conditions, treated with 30μM of R59949 and processed as in B. One representative experiment of $n \geq 3$ (A, B, C).

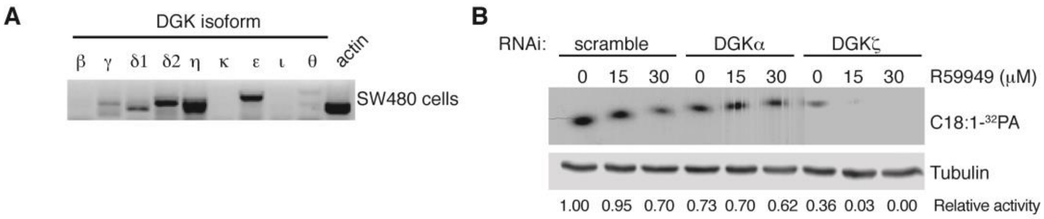


**Figure S3:** (A) Expression of the indicated DGK isoforms was determined by RT-PCR in SW480 cells. (B) SW480 cells were transfected with the indicated siRNA. After 72 h post-transfection, cells were treated with the DGK inhibitor for 24 h and the total DGK activity was evaluated. Tubulin was used as loading control. Relative DGK activity is indicated below autoradiograph. Activity of scramble, vehicle-treated cells = 1.00 ($n \geq 3$).

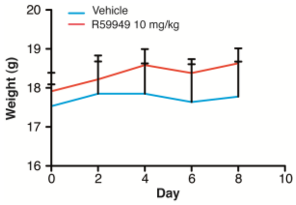


**Figure S4:** Record of weight of the mice injected with vehicle or R59949 along the treatment time.

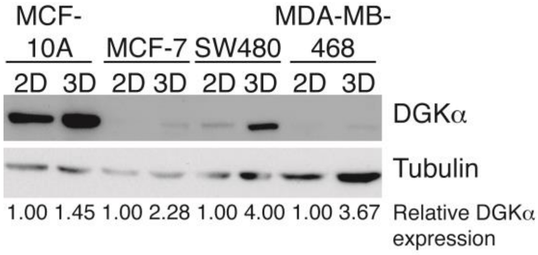


**Figure S5: DGKα levels increase in cells cultured in matrigel.** The indicated cell lines were plated on subconfluence (2 days) or on matrigel (6 days), and DGKα levels were analyzed by western blot. Tubulin was used as loading control. DGK levels were normalized to those of tubulin. A representative experiment is shown ($n = 3$).

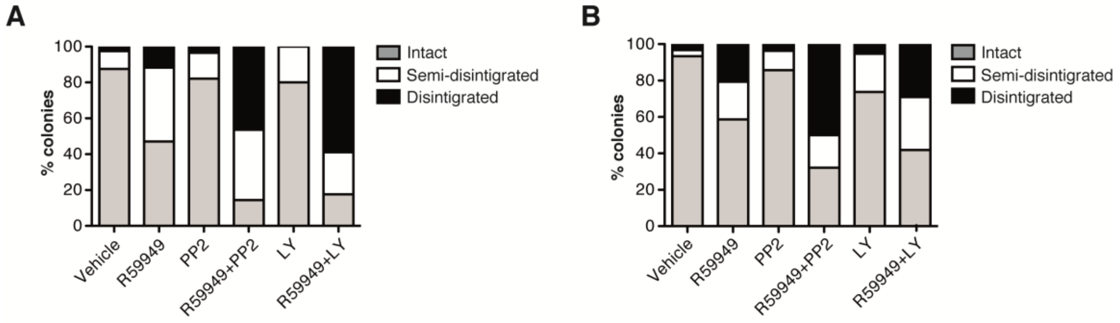


**Figure S6: Distribution of the different integrity patterns of 3D cultured cell colonies treated with different inhibitors.** SW480 (A) or MDA-MB-468 (B) cells were cultured in matrigel (4 days) and treated with the indicated drugs (48 h). Cell colonies were classified into three groups according to the integrity of the cells in intact, semi-disintegrated and disintegrated. Several fields from different experiments were examined and the values expressed as percentages.

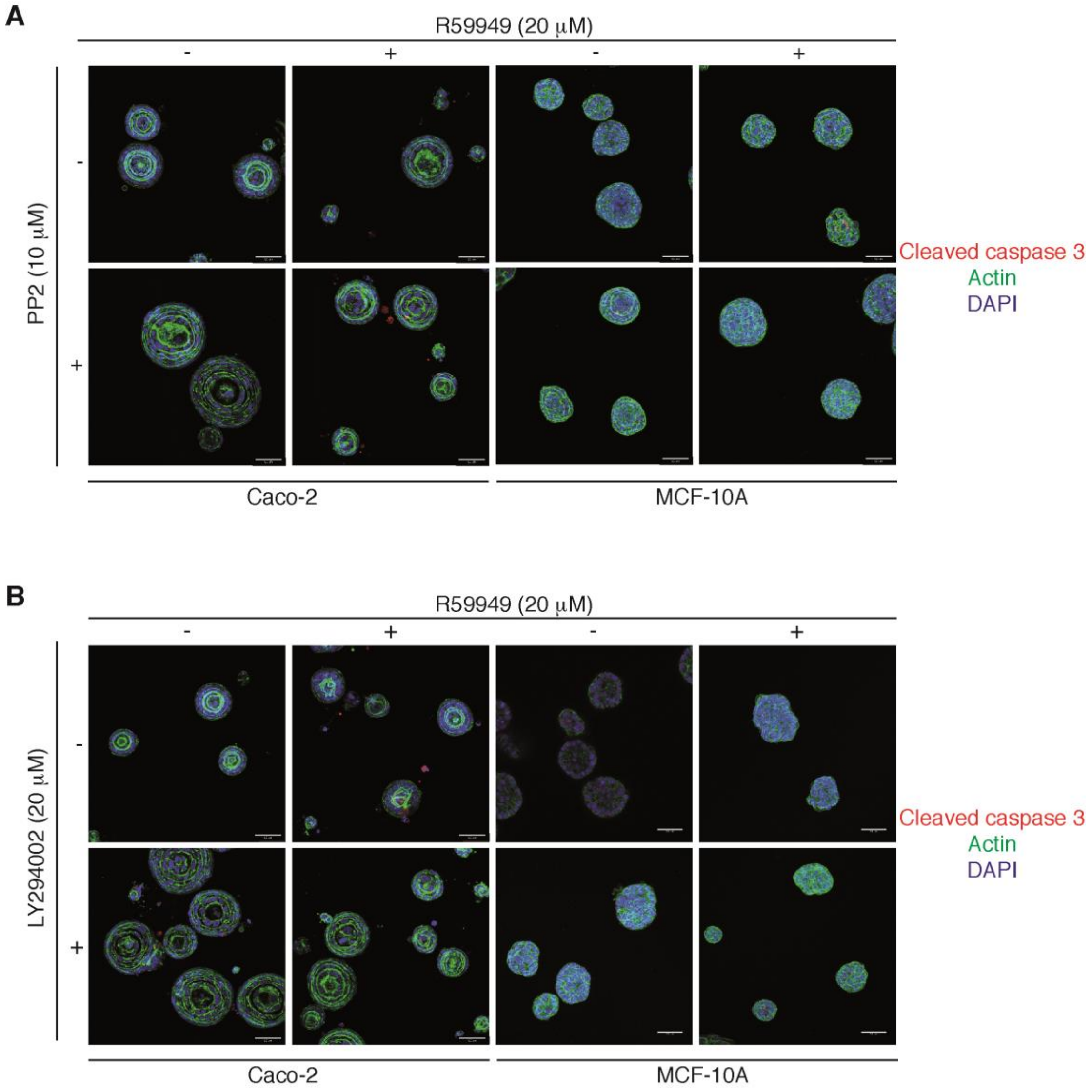


**Figure S7: DGKα and Src or PI3K inhibition did not promote apoptosis in 3D cultures of Caco-2 and MCF-10A cells.** (A) The indicated cells were seeded on matrigel (4 days), and then treated with either R59949, PP2 or both for 48 h. Cells were fixed, and apoptosis evaluated as cleaved caspase 3 staining. Actin and DAPI staining was also performed. (B) As in A, but cells were treated with R59949, LY294002 or both for 48 h. A representative experiment, of at least 3, is showed in the panels.
